# Infrasound exposure promotes development of atrial fibrosis in rats

**DOI:** 10.1080/07853890.2021.1897455

**Published:** 2021-09-28

**Authors:** Ana Lousinha, Maria João R. Oliveira, Gonçalo Borrecho, José Brito, Pedro Oliveira, Gonçalo Pereira, António Oliveira de Carvalho, Diamantino Freitas, Artur P. Águas, Eduardo Antunes

**Affiliations:** a Center for Interdisciplinary Research Egas Moniz (CIIEM), Health Sciences Institute, Monte de Caparica, Portugal; b Department of Anatomy and UMIB of Abel Salazar Biomedical Sciences Institute, University of Porto, Porto, Portugal; c Engineering Faculty, Polo da Asprela, University of Porto, Porto, Portugal

## Abstract

**Introduction:**

Recent data has shown a significant association between noise exposure and atrial fibrillation (AF) in a large cohort [1] but the pathophysiology remains unclear. The acoustic spectrum of industrial environments is particularly rich in high-intensity infrasound (IFS), which we have previously found to induce coronary perivascular fibrosis in rat hearts [2–4]. The role of atrial fibrosis in AF is well documented and remains the cornerstone of atrial pathology in patients with this arrhythmia [5]. The aim of this study was to evaluate and measure the atrial interstitial fibrosis in rats exposed to high-intensity IFS.

**Material and methods:**

Twelve Wistar rats exposed to high-intensity IFS (110 dB, <20Hz) during a period of 6 weeks and 12 age-matched controls were studied. All the handling and care of the experimental animals was performed by authorised researchers and was done in accordance with the EU Commission on Animal Protection for Experimental and Scientific Purposes (2010/63/EU). Hearts were transversely sectioned and the atrial fragment was selected for analysis. Chromotrope-aniline blue staining was used for histological observation and the images were obtained with an optical microscope using 400× magnifications. For each atrium, three optical fields containing more prominent fibrotic development in the absence of any arterial vessel were selected. The measurement of fibrosis was performed using *Image J software*. Mann–Whitney test was used to compare the groups.

**Results:**

The mean values of atrial interstitial fibrosis were 8.96 ± 4.08 and 4.91 ± 1.46, respectively, in IFS-exposed rats and controls. IFS-exposed rats exhibited a significant increase in atrial interstitial fibrosis (*p* = .005).

**Discussion and conclusion:**

High-intensity IFS induces atrial interstitial fibrosis in rats. This finding reinforces the need for further experimental and clinical studies concerning the effects of IFS on the heart.
